# Cryotherapy: A viable tool to remove broncholiths under flexible bronchoscopy

**DOI:** 10.7603/s40681-016-0024-2

**Published:** 2016-11-16

**Authors:** Sabrina N. Campbell, Deepa Lala, Edmundo Rubio

**Affiliations:** 1 Department of Pulmonary, Critical Care and Sleep Medicine, Carilion Clinic/Virginia Tech Carilion School of Medicine, 1906 Belleview Ave SE, Roanoke, VA 24014 USA; 2 Salem VA Medical Center, Salem, VA 24153 USA

**Keywords:** Broncholithiais, Broncholith, Cryotherapy, Lithoptysis

## Abstract

Broncholithiasis is the presence of calcific material within the tracheobronchial tree. Asymptomatic patients can be managed with observation only, whereas symptomatic disease requires surgery, rigid or flexible bronchoscopic removal. Recent reports have shown that flexible bronchoscopy can be a safe and effective option for removal of loose in addition to partially imbedded broncholiths. We present a case of a 65-yearold man with chronic cough that underwent successful cryotherapy assisted bronchoscopic removal of an imbedded broncholith. We will also review current literature regarding the management broncholithiasis.

## 1. Introduction

Broncholithiasis is the presence of calcific material within the tracheobronchial tree. Patients with this condition may be asymptomatic or have non-specific symptoms such as cough, wheezing, fever, chest pain and rarely lithoptysis. Asymptomatic patients can be managed with observation only, whereas symptomatic disease requires surgery, rigid or flexible bronchoscopic removal, sometimes assisted by laser photocoagulation and more recently through use of cryotherapy. We present a case of successful cryotherapy assisted bronchoscopic removal of an imbedded broncholith.

## 2. Case

**Fig. 1 -:**
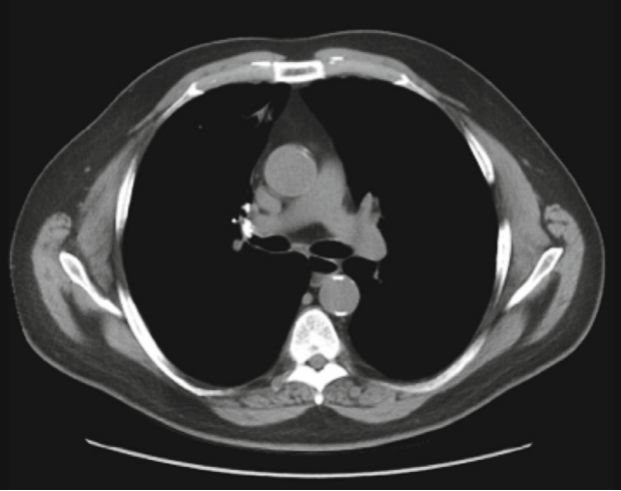
**Axial image of computed tomography of the chest showing the suggestion of a broncholith in the anterior RUL bronchus.**

**Fig. 2 -:**
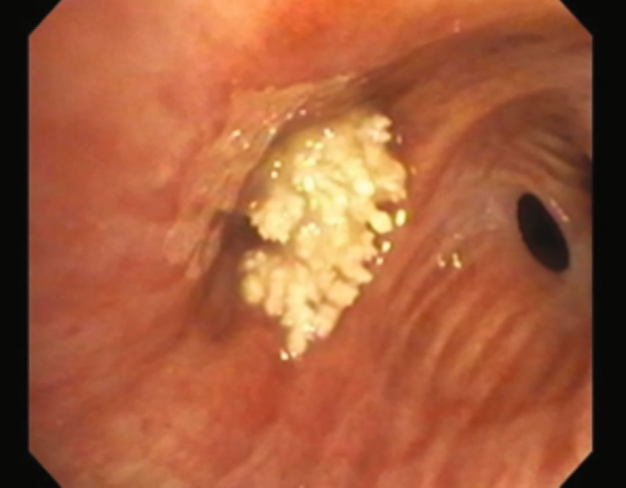
**Bronchoscopy image demonstrating at the entry to the RUL a calcific endobronchial tumor obstructing the anterior segment.**

**Fig. 3 -:**
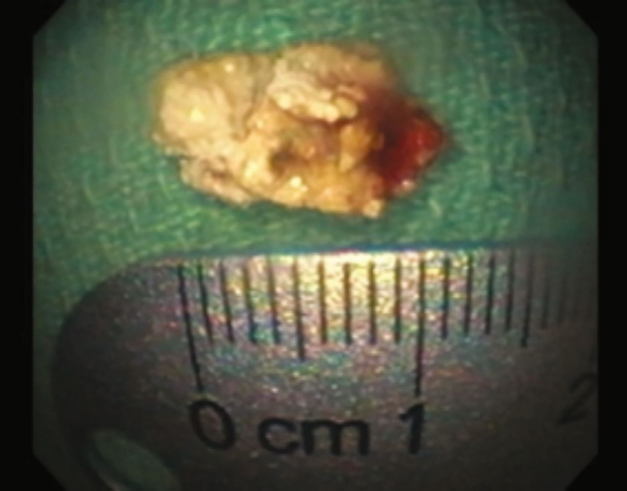
Broncholith post removal measuring 10 mm by 6 mm.

**Fig. 4 -:**
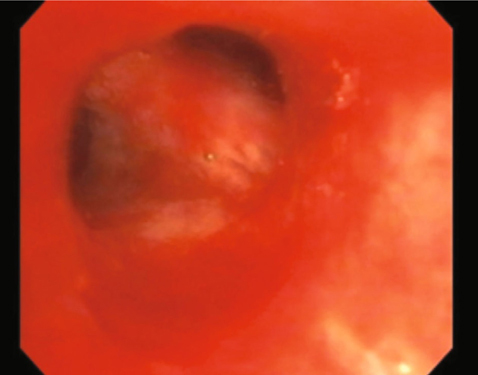
**Bronchoscopy images post removal demonstrating a now patent RUL anterior segment.**

A 65 year old man with a history of tobacco abuse, Agent Orange exposure, childhood asthma and obstructive pulmonary disease was seen in the pulmonary clinic for evaluation of chronic cough productive of yellow sputum for several years. Removal of triggers improved symptoms some, leading to a suspected diagnosis of upper airway cough syndrome. Therefore, he was treated with flunisolide nasal spray, plus ranitidine for suspected GERD, while continuing inhaled formoterol/mometasone in addition to tiotropium for his underlying obstructive lung disease. Nevertheless, he returned with persistent cough, subsequently undergoing high resolution computed tomography (HRCT) of the chest, which noted focal right upper lobe (RUL) bronchiectasis, reticular nodular changes and suggestion of a broncholith in the anterior RUL bronchus [Figure 1]. The patient did not follow up for results and after multiple attempts to contact him, he was reached and reported modest improvement in the cough. One year later he returned reporting worsening cough. Repeat chest computed tomography showed progression of the previous noted findings. He underwent bronchoscopy, demonstrating at the entry to the RUL a calcific endobronchial tumor obstructing the anterior segment [Figure 2]. Under anesthesia with use of a supraglottic airway and Olympus BF-1T180 therapeutic bronchoscope (Center Valley, PA) the calcific endobronchial tumor was removed using a 1.9 mm ERBE cryotherapy probe (Marietta, GA). The tumor was frozen for 6 seconds at its tip avoiding touch of the normal surrounding mucosa and pulled en-block with the bronchoscope removing the great majority of the tumor. The tumor measured 10 mm by 6 mm [Figure 3]. Upon re-examination there was a very small amount of residual partially embedded calcific tumor (around 2-3 mm in diameter) noted on the medial aspect of the airway mucosa in the region of the previously removed tumor. The cryotherapy probe was again utilized but with only a 3 second freeze time and again we removed en-block the residual tumor. There was no significant bleeding or any mucosal damage noted [Figure 4]. Pathology showed mucocalcific material consistent with a broncholith. The patient failed to return for follow-up 4 weeks later, but did present again 5 months later in pulmonary clinic. He reported his cough had significantly improved. Repeat CT chest no longer showed the obstructing RUL broncholith. Repeat bronchoscopy noted the RUL anterior segment to be patent. There was no recurrence of previously removed broncholith and only a white discoloration of the airway mucosa at the site, presumed to be a scar.

Broncholithiasis refers to calcified or ossified material in the lumen of the tracheobronchial tree. The most common postulated mechanism of formation is erosion and later extrusion into the bronchial lumen of a calcified necrotic bronchopulmonary lymph node [1]. The calcification is felt to occur after an inflammatory process, commonly following granulomatous inflammation due to Mycobacterial or fungal infections [2-4]. There are also rare reports of silicosis causing broncholithiasis [5, 6].

Patients may be asymptomatic, but when symptoms are present they are nonspecific and can include; cough, hemoptysis, chest pain, wheezing, sputum production and fever. Lithoptysis has also been reported [7, 8]. Complications of broncholithiasis include massive hemoptysis, bronchoesophageal fistulas and aortotracheal fistulas [9-11]. Recurrent or post obstructive pneumonias are also a known complication of broncholithiasis [7, 8, 12].

Imaging is abnormal in patients with broncholithiasis. In a case series [7] of 15 patients, both radiograph and computed tomography of the chest were abnormal. On radiograph, abnormalities included calcified lymph nodes near a bronchus, atelectasis, calcified granulomas or pulmonary infiltrates. CT features included atelectasis in patients with peribronchial or endobronchial lymph nodes, bronchiectasis distal to the obstructing lymph node, dense consolidation or patchy parenchymal infiltrates. Air trapping, though rare, is described. Lim *et al* [13] also noted additional imaging findings that included airway distortion and mucoid impaction. Most cases of broncholithiasis involve the right bronchial tree [13-15]. Bronchoscopy can be used as a diagnostic tool for classification of broncholiths (intraluminal, extra luminal or mixed) and for ruling out a lesion due to a calcified malignancy, which could be mistaken as a simple broncholith [13, 16]. Bronchoscopic findings can include bleeding, airway narrowing, extrinsic compression, stone like material in the airway lumen and anthracosis [13]. The bronchoscopic sensitivity to detect broncholiths is reported at 56% in one case series [7]. Sensitivity remains low, as the nodes have been noted to be peribronchial, obscured by blood and in addition they may be hidden by bronchial distortion due to edema and inflammation.

There are no current guidelines for the management of broncholithiasis. Therapeutic considerations include; size, location, degree of attachment to bronchial walls and association with vascular and mediastinal structures [11, 15]. Historically, surgical intervention was the preferred treatment [17] for all. Later it was felt that endoscopic removal should be attempted first, with thoracotomy reserved for patients with complications due to the broncholith [18]. Indications for surgical treatment include symptoms, persistent or massive hemoptysis, bronchiectasis, bronchial stenosis, stenosis and suppurative lung disease [17, 19-21]. Surgical options for removal include segmentectomy, open thoracotomy and lobectomy [13, 21].

Removal of broncholiths can also be safely accomplished by flexible or rigid bronchoscopy [13]. Nollet *et al* [8] utilized rigid bronchoscopy with use of neodymium-doped yttrium aluminum garnet (Nd-YAG) laser photocoagulation. Laser techniques can be utilized to shatter large stones that obstruct part of the airway. Further, treatment of the surrounding granulation tissue with laser photocoagulation therapy in patients with obstructive symptoms, even without the removal of the broncholith, can improve symptoms [3]. Previously thought of as unsafe and near impossible to remove imbedded broncholiths with flexible bronchoscopy [6, 8], recent reports have shown that flexible bronchoscopy can be a safe and effective option for removal of loose in addition to partially imbedded broncholiths [8, 14, 15]. Olsen *et al* [15] noted a 100% (23 of 23) and 46% (48 of 104) success rate of removal of lose and partially eroding broncholiths respectively. It was concluded that the degree of attachment was a factor in successful bronchoscopic removal, but unfortunately this report does not describe the specific techniques utilized to remove these broncholiths, with no mention to use of cryotherapy. Nevertheless, they do state that use of rigid bronchoscopy for removal was noted to have a higher rate of complete removal than using the flexible bronchoscope (67% *vs.* 30%). Reported complications of bronchoscopic removal include massive hemoptysis and complications due to loss of the broncholith in the trachea [15]. On review of 46 patients, Lim *et al* [13] concluded that surgery should be considered for extraluminal and mixed broncholiths, while bronchoscopy can be utilized to remove intraluminal broncholiths. In 2007 Reddy *et al* [4] reported the first case of broncholith removal using cryotherapy during flexible bronchoscopy with only minimal bleeding noted. They concluded that cryotherapy could be used to remove broncholiths that cannot be extracted with a flexible forceps. In 2012 Lee *et al* [22] also performed cryotherapy-assisted removal of broncholiths in two patients. One was performed using the repeated freeze-thaw method while in the other case the broncholith was frozen and the pull-out method was utilized. The case in which the freeze and pull-out method was performed did require Argon Plasma Coagulation (APC) to control minor bleeding. Our case employed a freeze pull-out method, without damage to the mucosal wall or significant bleeding. When considering bronchoscopic removal of a broncholith, capabilities for rigid bronchoscopy should be available [15], which was also immediately available in our case. Massive hemoptysis is considered a contraindication for bronchoscopic removal and complicated cases of broncholithiasis should be managed surgically [14]. As such, immediate thoracic surgical support should also be available at the time of bronchoscopy. Additionally, the time of freezing and feasibility to remove the broncholith with a pull-out method requires experience and the ability to assess that the retracting force is not too excessive, such as to damage the wall by pulling on a broncholith that is too embedded in the bronchial wall. The freeze time before attempting to pull should be in direct proportion to the size of the lesion and its proximity to normal mucosa. As noted, we used two different times in our case, longer (6 seconds) in our initial attempt and shorter (3 seconds) to remove the residual broncholith fragment.

Finally, the decision to pursue a bronchoscopic approach should also include careful examination of the chest tomography images and the relation of the broncholith with surrounding structures, particularly vessels.

## 3. Conclusion

While bronchoscopic removal of broncholiths using instruments such as forceps, balloon catheters, snares and baskets appears a viable option in both loose and partially imbedded broncholiths, cryotherapy appears to be a good and perhaps easier alternative to remove these lesions. In fact, in candidates deemed appropriate, this could be the initial approach prior to referral for surgical intervention. Further studies in this area are encouraged.
